# Key External Influences Affecting Consumers’ Decisions Regarding Food

**DOI:** 10.3389/fpsyg.2016.01618

**Published:** 2016-10-18

**Authors:** María Pilar Martínez-Ruiz, Carmen María Gómez-Cantó

**Affiliations:** Facultad de Ciencias Económicas y Empresariales, Plaza de la UniversidadAlbacete, Spain

**Keywords:** food products, food values, consumer research, decision making

## Abstract

Among the numerous internal and external forces that compete for consumers’ attention in the context in which they buy their food, this paper will seek to provide a review of the most important external influences, such as the variables related to food itself. To this end, in addition to the food attributes traditionally identified in fields such as consumer behavior, it will give special consideration to the classification of food values. Although the influence of these variables on consumer decisions depends on the individual, analyzing them will undoubtedly increase understanding of consumers’ decisions. Additionally, identifying and describing these variables will enable subsequent research on how they influence both consumer behavior and other key outcomes for producers, manufacturers, and retailers in the food industry, such as satisfaction, trust, and loyalty.

## Introduction

Recent decades have witnessed a number of changes in the buying habits and behaviors consumers have traditionally shown when purchasing food products (Pieniak et al., 2010; De Moura et al., 2012; Deloitte, 2015). To understand these changes, it is necessary to take into account the large number of forces, both internal (e.g., prior experience) and external (e.g., characteristics of the food products themselves), that compete for consumers’ attention in the context in which they make their decisions (c.f. Garber et al., 2003; Mowen and Minor, 2003; Logue, 2015). Although the extent to which these influences ultimately affect consumers’ buying behavior will depend on the individual, analyzing them will undoubtedly increase understanding of consumers’ purchase decision processes with regard to food and, thus, facilitate proper planning for producers, manufacturers, and retailers in the food industry.

Consumer research is particularly difficult for food, among other things, because of the especially subtle and complex nature of food products as stimuli at points of purchase and during consumption (Garber et al., 2003). Consequently, among other external influences to receive attention, the relevant literature has focused in particular on variables related to food products themselves (Garber et al., 2003; Lusk and Briggeman, 2009; Lusk, 2011). Unsurprisingly, there is thus increasing interest in the field in trying to identify which food-related variables exert the strongest influence on consumer behavior (Deloitte, 2015; Logue, 2015).

Drawing on these ideas, this paper offers a synthetic review of those food variables that the relevant literature has identified as key external influences, including the latest developments. Specifically, it will address the research on food attributes and the food values identified by Lusk and Briggeman (2009), a significant development in the research in this field. The identification and description of these variables is of great interest to enable subsequent work in this area, since: (i) it sheds light on which food variables have generally been considered key and should be taken into consideration in future research; and (ii) it facilitates the subsequent analysis of potential influences on and interrelations with various stages of the food-purchase decision process, as well as on other key results for retailers in the consumer goods industry, both financial (sales and profitability) and non-financial (satisfaction, trust, and loyalty).

## Food Variables as External Influences in the Consumer Decision Process: A Literature Review

In recent decades, numerous studies have sought to measure consumer preferences for certain food attributes over others (Lusk and Briggeman, 2009). However, some more recent work, such as that by Lusk and Briggeman (2009), has moved beyond the food *attributes* traditionally considered in the literature to propose a classification of food *values*, that is, a stable set of beliefs regarding the relative importance of the meta-attributes, consequences, and desired end states associated with purchasing and consuming food.

In light of the importance of both food attributes and food values as external influences affecting consumers’ purchase decision processes with regard to food, the following sections will first review the food attributes generally considered in the relevant literature, in order to then use that discussion as a starting point to describe food values.

### Product Attributes

Product attributes provide a basis both for marketers to differentiate and position existing products apart from those of their competitors and for the development of new products. This may be done based on a specific attribute or range of attributes or on several attributes at once (Belch and Belch, 1995; González-Benito et al., 2010).

A product’s attributes influence consumers’ product choices and are able to play a variety of roles (informational, communicative, symbolic, etc.). Industry operators must know what value consumers attach to those attributes, as well as how they factor into the purchase decision process. Moreover, companies must endow their products with the right level of attributes to meet consumers’ expectations, without neglecting related managerial decisions, usually involving resource-allocation, cost, and price-setting considerations. Also, although decades ago the earliest work in the field tended to take into account only quantifiable product attributes that were objectively measurable, such as price, more recently, researchers have begun to include more subjective attributes in their work, such as quality (e.g., Kotler and Keller, 2012).

This broader and relatively more recent view of product attributes including attributes that are not only of an objective and measurable nature is clearly on display in the literature on food products. Indeed, until fairly recently, when choosing a given food product, consumers barely considered other types of issues, such as those related to good farming practices, food safety during the production process, nutritional quality, or the convenience or ease with which the product could be prepared and consumed (Berné and Martínez, 2007). In contrast, today’s consumers have more and more information on these aspects and are thus more demanding when choosing the food they want to purchase. For instance, Robinson (2002) found that consumers supported sustainably produced food, although, paradoxically, they were not particularly likely to purchase it.

Nutritional aspects also generate considerable interest among end consumers, influencing their food choices. Consumers use this information to determine what nutrients they ingest, which largely affects their health (Kissileff and Van Itallie, 1982). In this regard, in their analysis of consumer orientations toward the health and hedonic characteristics of food products, Roininen et al. (1999) identified three health-related factors—general health interest, light product interest, and natural product interest—and three taste-related factors—craving for sweet foods, using food as a reward, and pleasure. They also found that women were more interested in health- and taste-related aspects than men, and that young people were less concerned with health and more interested in taste than older consumers.

Recent research by Deloitte (2015) confirmed the growing relevance of health-related attributes to consumers’ food purchase decisions, noting that taste, price, and convenience are no longer the sole drivers of consumers’ food and beverage purchases. The study further found that, in addition to these traditional drivers, more than half of American consumers now weigh the following drivers in their purchase decisions too: health and wellness, safety, social impact, experience, and transparency.

Food buying and consumption behavior has been widely studied from a psychological perspective, making it possible to focus on certain attributes over others (Logue, 2015). For instance, focusing on food reward, Berridge (1996) found that food consumption may be influenced by, among other things, certain taste-related psychological aspects, derived from the pleasure of the act of eating and the pleasantness of a food’s taste.

Other lines of research in this area conducted from a psychological perspective include (Logue, 2015): (i) how people detect flavors; (ii) why people prefer some food to others; (iii) how people end up choosing certain foods over others; and, more specifically, (iv) how and why certain foods are able to influence consumers’ choice behavior. All have considered numerous and diverse food attributes, to which they assign varying degrees of importance. Finally, from a perspective that transcends psychology, food provides people with opportunities to communicate and engage in a variety of socialization processes, allowing them to express and maintain their lifestyles, which are often linked to their individual cultures (Atkins and Bowler, 2001; Logue, 2015).

### Food Values

To understand how consumers evaluate food attributes and how they impact in the purchase decision process, it is particularly relevant to consider the article of Lusk and Briggeman (2009), who, in a key contribution, moved beyond the simple consideration of traditional food attributes. Although this work was published in the area of agricultural economics, the proposed values were developed based on a profound literature review on food preferences and human values, which enables to recognize these values as a cornerstone contribution in fields such as marketing and consumer behavior. Without doubt these values are closely related to the advent of the values-driven era (Marketing 3.0), that highlights the need to take care of customers not as mere consumers but as complex and multi-dimensional human beings with minds, hearts, and spirits. Because under this philosophy, customers choose those companies and products that satisfy their deepest needs for economic, social, and environmental justice (Kotler et al., 2010).

Rather than estimating consumers’ preferences for certain specific food products and attributes, which consumers might have little knowledge of and/or experience with, Lusk and Briggeman (2009) identified a set of food values or meta-attributes for which people might have better-defined preferences in order to gain greater insight into why consumers choose certain food products or attributes over others. To this end, these authors conducted a thorough review of the relevant literature on consumers’ willingness to pay for food products and human values, which allowed them to apply the concept of overall life value, previously defined in pioneering work such as Rokeach (1973) and Schwartz (1992), to food. They were thus able to identify a set of food values likely to remain relatively stable over time. The aim was not merely to identify food attributes per se, but rather to identify more abstract attributes, consequences and end states of food consumption that might be applied to explain consumers’ choices between a wide range of foods.

Specifically, they identified the following values (Lusk and Briggeman, 2009):

• Naturalness, i.e., the extent to which food is produced without modern technologies;
• Taste, i.e., the extent to which consumption of food is appealing to the senses;
• Price, i.e., the amount paid for food;
• Safety, i.e., the extent to which consumption of food will not cause illness;
• Convenience, i.e., the ease with which food is cooked and/or consumed;
• Nutrition, i.e., the amount and type of fat, protein, vitamins, etc., food contains;
• Tradition, i.e., the preservation of traditional consumption patterns;
• Origin, i.e., where the agricultural commodities were grown;
• Fairness, i.e., the extent to which all parties involved in food production benefit equally;
• Appearance, i.e., the extent to which food looks appealing;
• Environmental impact, i.e., the effect of food production on the environment.

As Lusk and Briggeman (2009) explain, although some of these values may initially seem very similar to some of the classically considered food product attributes, they represent more abstract concepts, often encompassing numerous physical attributes at once. For instance, the value of nutrition can be considered more stable than a consumer’s relative preferences for specific vitamin or fat contents. Likewise, while some of the proposed values, such as price, can be classified as personal (i.e., self-centered), others, such as tradition, origin, fairness, and environmental impact, can be regarded as social (i.e., society-centered).

To determine the relative importance consumers give to these attributes, Lusk and Briggeman (2009) conducted a survey of consumers in the US. They found that: (i) in general, there was significant heterogeneity across consumers in terms of the relative importance they assigned to food values; and (ii) on average, safety, nutrition, taste, and price were among the values consumers considered most important.

In a subsequent study, Lusk (2011) found that food values are significantly related to actual grocery store purchases, suggesting that the food values scale could potentially be used, among other things, to explain consumer choice and guide new product development and marketing decisions.

## Discussion

This paper has offered a review of those food variables that, acting as external influences, impact consumers’ food-purchase decision processes. To this end, it first addressed the food attributes traditionally considered by the relevant literature, showing that, although early research tended to focus especially on objectively measurable attributes, more recently there has been a growing trend toward including more subjective ones. Indeed, recent data have confirmed the need to include these latter types of attributes, as, in addition to assessing easily quantifiable and objective attributes such as price, consumers increasingly also weigh other attributes in their decisions, such as those related to health or wellness.

This paper also considered the eleven food values identified by Lusk and Briggeman (2009), a significant advance in the research in this field. These authors suggest that consumers have intermediary values, consisting of a stable set of beliefs regarding the relative importance of the meta-attributes, consequences, and desired end states associated with food purchases. These values are intended to represent the main values influencing food choice and be comprehensive enough to cover the full breadth of issues that tend to drive consumers’ decisions with regard to food.

The synthesis provided in this paper helps pave the way for future research in the field by identifying the food variables that are generally considered to be important and should thus be taken into account. This will facilitate their inclusion in subsequent studies, for instance, on potential influences and interrelationships in different stages of the food-purchase decision process or with regard to other key outcome variables.

Finally, one limitation of this article lies in the small number of external influences considered. Future research should thus expand the review to include other external factors, as well as address internal influences.

## Author Contributions

All authors listed, have made substantial, direct and intellectual contribution to the work, and approved it for publication.

## Conflict of Interest Statement

The authors declare that the research was conducted in the absence of any commercial or financial relationships that could be construed as a potential conflict of interest.
